# Living on the edge: morphological, karyological and genetic diversity studies of the Hungarian *Plantago maxima* populations and established ex situ collection

**DOI:** 10.1186/s40529-022-00365-6

**Published:** 2023-01-24

**Authors:** Zsófia Kovács, Jelena Mlinarec, Mária Höhn

**Affiliations:** 1 Institute of Agronomy, Department of Botany, Hungarian University of Agriculture and Life Sciences, Villányi Út 29-43, Budapest, 1118 Hungary; 2 Department of Zoology, Plant Protection Institute, Centre for Agricultural Research, ELKH, Budapest, Hungary; 3 Department of Nature Protection and Landscape Architecture, Oikon Ltd.-Institute of Applied Ecology, Trg Senjskih Uskoka 1-2, 10020 Zagreb, Croatia

**Keywords:** Chromosomes, Conservation, FISH, Giant plantain, ISSR, Morphometric traits, trnL-trnF

## Abstract

**Background:**

The analysis of genetic diversity of protected plant species can greatly support conservation efforts. *Plantago maxima* Juss. ex Jacq. is a perennial species distributed along the Eurasian steppe. The westernmost range edge of the species’ distribution is located in the Pannonian basin, in Hungary where it is represented by a few, fragmented and highly endangered populations. We studied population diversity of all Hungarian range edge, natural populations, and one established ex situ population. One population from the centre of distribution (Kazakhstan) was implemented in the cpDNA haplotype study to compare the peripheral vs. central populations. We performed morphometric trait-based analysis, chromosome studies (morphometric analyses and FISH) and genetic diversity evaluations using inter simple sequence repeats (ISSR) and cpDNA trnL-trnF to evaluate differences between the in situ and ex situ populations as well as central vs. peripheral populations.

**Results:**

Our results showed no obvious morphological differences among the in situ and ex situ populations in the period between 2018 and 2020. One ex situ subpopulation develops flowers three years in a row from 2019, which is a favourable indicator of the introduction success. Hungarian populations are exclusively diploids (2*n* = 2x = 12). The karyogram consists of 5 metacentric and 1 acrocentric chromosome pair. *Plantago maxima* has one 35S and two 5S rDNA loci, located on the acrocentric chromosome pair. Eight variable ISSR primers yielded 100 fragments, of which 74.6% were polymorphic (mean H_e_ = 0.220). A high level of genetic variation within population was observed (92%) while the genetic differentiation among the populations was only 8%. STRUCTURE analysis revealed that the largest Kunpeszér population separated from the rest of the Hungarian populations, indicating a high rate of admixture among the other ones. Based on the trnL-trnF sequence analysis the Hungarian populations represent a single haplotype, which can indicate a reduced diversity due to isolation and recent population decline. By contrast, Kazakh population represents a distinct haplotype compared to the Hungarian samples.

**Conclusions:**

The present study draws the attention to the high conservation value of the *Plantago maxima* populations from the westernmost range edge of the species’ distribution.

**Supplementary Information:**

The online version contains supplementary material available at 10.1186/s40529-022-00365-6.

## Background

It has been emphasized since long time that preserving genetic diversity of endangered species can significantly affect the long-term survival and evolution in changing environments (Frankham 2003). In situ or on-site conservation is to protect, manage and monitor a target species’ population within its natural habitat (Heywood 2014). Ex situ conservation is the other possible tool for the preservation of endangered and rare plant species. One of the primary goals of this method is to preserve genetic variation and representativeness outside the species’ native habitat (Maunder and Byers 2005; Volis and Blecher 2010). Moreover efficient ex situ conservation strategies require information on the genetic variation and structure of the target species (Brown and Briggs 1991; Brown and Marshall 1995; Pupin et al. 2019) supplemented with adaptive traits variation (Volis and Blecher 2010). Studies on population diversity and genetic structure of endangered species are therefore urgently needed to promote effective conservation and management activities (Wu et al. 2015).

The cosmopolitan *Plantago* genus (*Plantaginaceae*) is 5–18.5 Million years old (Rønsted et al. 2002; Cho et al. 2004; Iwanycki Ahlstrand et al. 2019) and comprises over 250 species which are distributed in the temperate and high-elevation tropical regions (Rahn 1996; Rønsted et al. 2002; Li et al. 2011). One of the strictly protected species growing in Hungary is the perennial giant plantain (*Plantago maxima* Juss. ex Jacq). *Plantago maxima* is the member of subg. *Plantago* and sect. *Lamprosantha* (Hassemer et al. 2019; Mower et al. 2021). The species has a Eurasian continental distribution (Soó 1968; Vidéki and Máté 2003). The range of the species extends through Eastern Europe to West Asia (Grigoriev 1958; Vidéki and Máté 2003). In Europe beside the Hungarian populations the species has one more isolated locality in Bulgaria (Tzonev and Karakiev 2007). With its four remaining and isolated peripheral populations in Hungary the species reaches the westernmost limit of its distribution area (Vidéki and Máté 2003; Kovács et al. 2018). The increasing fragmentation of the natural populations in Hungary and populations’ decline was induced in the twentieth century by the water regulation, intensified agricultural land use, herbal overcollection and fires resulting from military firing exercises (Vidéki and Máté 2003; Molnár-Baji 2013; Kovács et al. 2018). All these impacts threaten the long-term survival of the peripheral giant plantain populations. The Kunpeszér population is the largest, consisting approx. 2000 individuals located in the site of the Kiskunság National Park Directorate. The other three populations (Kakucs, Táborfalva and Tatárszentgyörgy) that belong to the Danube-Ipoly National Park Directorate are smaller and are even more fragmented and isolated, comprising fewer individuals. Therefore, the establishment of ex situ collection was a high priority. Seeds collected from the Kakucs area in 2015 were used for the ex situ stock establishment. Germination was accompanied by biological and morphological studies (Kovács et al. 2018; Kovács et al. 2019). The ex situ site was selected according to the habitat type and species composition from where the seeds originated. Ex situ plantlets were introduced on the *Molinia* fen meadow of the Soroksár Botanical Garden in 2016.

Basic chromosome number of *Plantago* sp. is 4, 5 and 6 (Peruzzi and Cesca 2002; Dhar et al. 2006; Shahriari et al. 2018). The majority of *Plantago* species are diploids, however, tetraploids, hexaploids, octoploids, decaploids, dodecaploids, and 16-ploids are also reported (Dhar et al. 2006; Wong and Murray 2014). Previously, it was reported that *P. maxima* is tetraploid 2*n* = 4x = 24 (Magulaev 1982). In contrast Soó (1970) and Rahn (1996) reported diploid level (2*n* = 2x = 12) for *Plantago maxima* which emphasizes the need to evaluate the chromosome number of the living Hungarian populations.

Mapping of rDNA loci using fluorescence in situ hybridization can be used to determine phylogenetic relationships in the *Plantago* genus (Dhar et al. 2006). Until this study the chromosomal position of 35S and 5S rDNA loci has been investigated for fewer than 10 *Plantago* species, exhibiting from one to two 5S rDNA loci and from one to two 35S rDNA loci (Dhar et al. 2006, 2017; Wong and Murray 2014). rDNA loci number and position is so far unknown for *P. maxima*.

Inter-simple sequence repeat (ISSR) polymorphism requires no prior knowledge of the DNA sequence and evolve rapidly enough to exhibit variation, therefore ISSRs are universally applicable as dominant markers even for exploring new species (Sa et al. 2011; González-López et al. 2014).

ISSRs have been widely and successfully used for genetic mapping and for evaluating population genetic variation in different *Plantago* species. Five to 25 ISSR primers were used, and the average polymorphism resulted between 56.67 and 83.83% (De Vita et al. 2009; Ferreira et al. 2013; Rahimi et al. 2017; Osman and Abedin 2019; Bagheri et al. 2022).

Chloroplast DNA (cpDNA) markers are widely used for taxonomic and phylogenetic comparisons (Shaw et al. 2007). In turn ISSRs with high rate of polymorphism could lead to a better resolution at intraspecific level. While cpDNA sequences are more slowly mutating represent a different time horizon (Kropf et al. 2020). Due to the knowledge gap of the whole genome of *Plantago maxima* and lack of a priori knowledge of DNA sequences ISSR and cpDNA markers were chosen in our analysis.

The goal of this study was to (1) evaluate the morphological trait variation of the Hungarian populations and the genetic representativeness of the ex situ stock; (2) give detailed information about the chromosome number and rDNA localisation; (3) assess the level of genetic diversity of both in situ and ex situ Hungarian *Plantago maxima* populations; (4) evaluate the possible haplotype differences using a cpDNA marker between a central vs edge populations.

## Methods

### Study sites and plant material

**Fig. 1:**
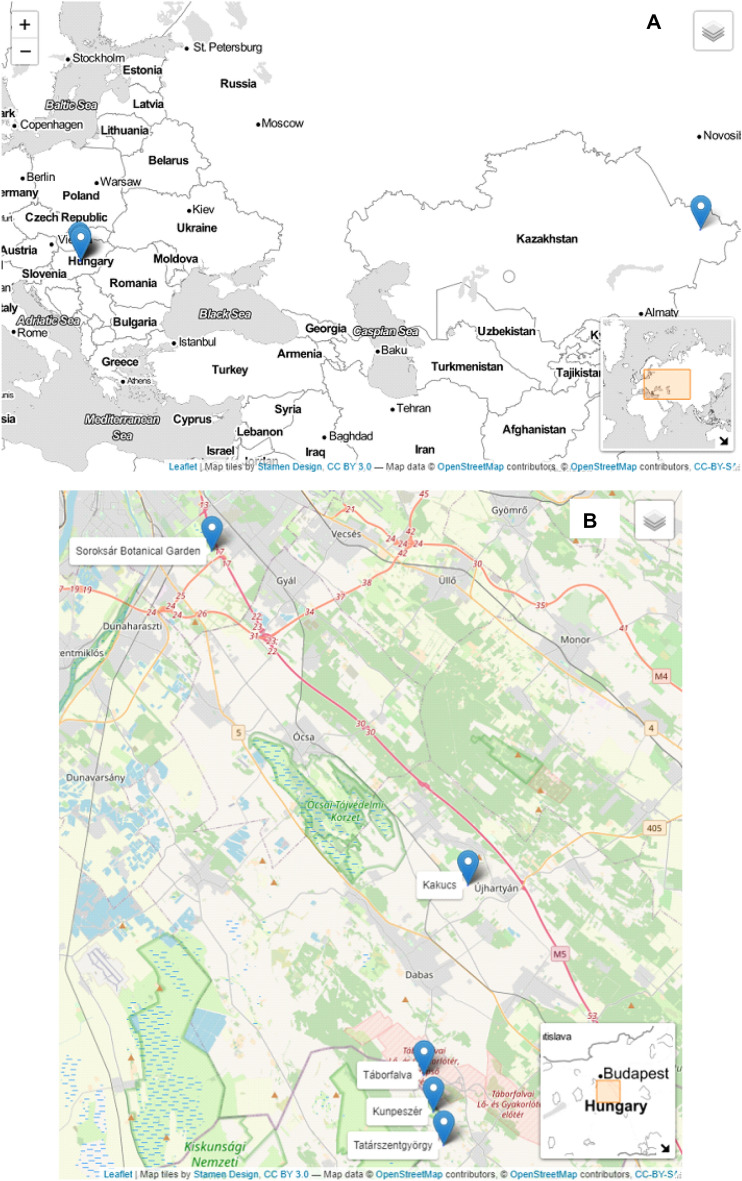
The map of the sampled populations of *Plantago maxima* in Hungary and Kazakhstan (**A**). The Hungarian localities are presented in better resolution **B** in a terrain map (source: https://rstudio.github.io/leaflet/)

Four in situ and one ex situ populations of *P. maxima* were included in the analysis from Hungary (Fig. 1). Morphometric measurements were conducted between 2018 and 2020 at the time of main flowering. The seed and leaf sampling for chromosome and genetic diversity studies was carried out in 2019 and 2020. The four in situ populations are located in *Molinia* meadows (Fig. 1). These sites are fragmented and are isolated from each other. The Soroksár Botanical Garden holds a natural *Molinia* meadow site, which proved to be optimal for the ex situ conservation of the giant plantain. Three ex situ subpopulations were established in 2016 and the habitat preference of the species was evaluated. Based on the species composition, and the water regime we observed differences among the three sites (Kovács et al. 2019).

One population from the central part of the species distribution range from Kazakhstan was implemented in the study, to compare population haplotypes between the edge and central locality. The received plant material was not applicable for morphometric, chromosome and ISSR study, therefore were only used for the cpDNA survey. Altogether four Hungarian and one Kazakh in situ*,* and one Hungarian ex situ populations of *Plantago maxima* were included in the cpDNA study (Fig. 1). The detailed information about the populations is listed in Table 1.

**Table 1:** Locations and information of the sampled *Plantago maxima* populations

Site	ID	Country	GPS coordinates	Elevation (m)	Number of individuals	n (morphometric analysis)	n (ISSR/trnL-trnF)
Kakucs	KA	Hungary	47.22094233 19.36152644	102	~ 250	20	20/4
Táborfalva military shooting range	LO	Hungary	47.11882594 19.32621506	99	~ 35	17	20/4
Tatárszentgyörgy	T	Hungary	47.08095949, 19.34209749	96	~ 30	20	20/4
Kunpeszér	KU	Hungary	47.099115, 19.334108	97	~ 2000	20	20/4
Soroksár Botanical Garden	EX	Hungary	47.400412, 19.158232	108	~ 60	13–20/subpopulation	20/4
Sibin depression	KH	Kazakhstan	49.431944, 82.610833	783	~ 50	N.E	N.E./4

### Morphometric characteristics and data analysis

In 2018–2020, yearly, morphometric parameters, individual growth of the in situ and ex situ populations and subpopulations were monitored. 13–20 individuals from the five Hungarian localities (4 in situ, 1 ex situ) were measured with a measuring tape. The observed traits were as follows: biggest leaf length, biggest leaf width, number of leaves. The generative parameters were not assessed, since the first flowers appeared in the ex situ stock in 2019, but in statistically non-significant quantities. However, the flowering individuals were monitored during the years to evaluate the background factors necessary for the development of generative organs.

Statistical analysis was carried out using IBM SPSS Statistics 27 software. Multivariate ANOVA (MANOVA) model was used to evaluate statistical differences between populations. Morphological traits as factors and years were treated as blocks in the model. Wilk’s lambda as unexplained variance rate was tested. Normality of the residuals was proved according to their skewness and kurtosis (D’Agostino et al. 1990; Tabachnick and Fidell 2013; p > 0.05). In case of the number of leaves normality was violated, therefore ln transformation (λ = − 0.3) was used. Homogeneity of variances was tested by Levene’s method. Populations were separated by Games–Howell’s or Tukey’s post hoc tests depending on whether homogeneity assumption was violated or not.

### Chromosome analyses and fluorescence in situ hybridization (FISH)

Seeds were collected from the four Hungarian in situ populations in 2019. Ploidy level from pooled seed samples, from 9 to 23 individuals per population were determined. Chromosome numbers were counted from mitotic metaphases from root tips using classical squash methods according to Mlinarec et al. (2006) with some modifications. Briefly, root tips were pretreated with ice-cold water at room temperature for 24 h, fixed in 3: 1 (v/ v) ethanol/acetic acid at 4 °C for 24–48 h and stored until use. Chromosomes were stained with antifade buffer Vectashield (Vector Laboratories, Peterborough, UK) containing DAPI counterstain (2 μg ml^−1^) and stored at 4 °C. Photographs were taken with an Olympus BX51 microscope, equipped with a highly sensitive digital camera (Olympus DP70).

Chromosome preparations for fluorescent in situ hybridization (FISH) were conducted according to Mlinarec et al. (2019). Briefly, the clone pTa794, containing the complete 410-bp BamHI fragment of the 5S rRNA gene and the spacer region of wheat (Gerlach and Dyer 1980), was used as the 5S rDNA probe. The 2.4-kb HindIII fragment of the partial 18S rDNA and ITS1 from *Cucurbita pepo*, cloned into pUC19 (Torres-Ruiz and Hemleben 1994), was used as the 35S rDNA probe. The hybridisation mixture (20 μL) containing 50% formamide, 10% dextran sulphate, 0.6% sodium dodecyl sulphate, 2 × SSC and 2 ng μl^−1^ of labelled probe was denatured at 96 °C for 3 min. Chromosome preparations denatured at 71 °C for 5 min after applying the hybridisation mixture. Stringent washes were performed at 42 °C in the following solutions: 2 × SSC, 0.1 × SSC, 2 × SSC, 4 × SSC⁄Tween (5 min each). The preparations were mounted in antifade buffer Vectashield (Vector Laboratories, Peterborough, UK) containing DAPI counterstain (2 ug ml^−1^) and stored at 4 °C until use. Signals were visualised and photographs captured with an Olympus BX51 microscope, equipped with a highly sensitive digital camera (Olympus DP70). Images were merged and contrasted using Adobe Photoshop 22.5.4. An average of 10 well-spread metaphases were analysed for each individual.

### DNA extraction and PCR amplification

Leaves were collected and stored in silica gel until use. DNA was extracted with E.Z.N.A.® SP Plant DNA kit (Omega Bio-tek, Inc., Norcross, GA, USA) following the manufacturer’s recommendations. DNA concentration and quality was assessed using NanoDrop (BioScience, Hungary).

### ISSR analysis and genetic structure

We performed 15.5 μL PCR reactions with 1 μL of genomic DNA, 10.72 μL of Milli-Q ultrapure water (Merck Millipore, Billerica, MA, USA), 1.5 μL of (10x) DreamTaq Green PCR buffer (ThermoFisher, Waltham, MA, USA), 0.3 μL of (10 mmol·L–1) dNTP mix (ThermoFisher, Waltham, MA, USA), 0.5 μL of (2.5 mmol·L–1) MgCl2, 0.5–0.5 μL (10 mmol·L–1) of each primer, and 0.03 μL of DreamTaq Green DNA polymerase (ThermoFisher, Waltham, MA, USA), 0.15 μL BSA (1%) (ThermoFisher) and 0.3 μL DMSO (2%). ISSR primers (Table 2) were chosen from the ISSR primer set 9 described at the University of British Columbia (http://www.michaelsmith.ubc.ca).

**Table 2:** The ISSR primer sequences and annealing temperatures applied in the *Plantago maxima* study

Primer ID	Sequence (5’ → 3’)	*T*a (°C)
UBC 807	5’-AGA GAG AGA GAG AGA GT-3’	49
UBC 808	5’-AGA GAG AGA GAG AGA GC-3’	49
UBC 809	5’-AGA GAG AGA GAG AGA GG-3’	49
UBC 811	5’-GAG AGA GAG AGA GAG AC-3’	49
UBC 816	5’-CAC ACA CAC ACA CAC AT-3’	49
UBC 818	5’-CAC ACA CAC ACA CAC AG-3’	49
UBC 835	5’-AGA GAG AGA GAG AGA GYC-3’	49
UBC 857	5’-ACA CAC ACA CAC ACA CYG-3’	49

*Ta* annealing temperature

Thermocycling conditions were as follows: initial denaturation at 94 °C for 5 min; followed by 40 cycles of 94 °C for 30 s, 49 °C for 60 s, 72 °C for 90 s; and a final synthesis at 72 °C for 7 min. Amplifications were performed with an Aeris™ Thermal Cycler (Esco Micro Pte. Ltd., Singapore). The PCR products were applied on a 1.5% (w/v) ethidium bromide stained agarose gel in 1xTBE buffer. PCR products were separated for 60–150 min at 110 V. Size comparison with external standards (Thermo Scientific GeneRuler 100 bp Plus DNA Ladder) was evaluated using GelAnalyzer 19.1 software (Lazar and Lazar 2010). Amplified fragments were scored visually for presence (1) or absence (0) of homologous bands and the results were summarized in MS Excel table. The binary data of ISSR markers were analyzed as dominant markers using GenAlEx 6.51b2. (Smouse et al. 2017).

The binominal ISSR data matrix was used to calculate a similarity matrix using Jaccard’s coefficients. Sequential agglomerative hierarchical non-overlapping (SAHN) clustering was employed using unweighted pair group method with arithmetic averages (UPGMA) method. Cluster analysis based on Nei’s genetic distances calculated in GenAlex was also carried out using the (UPGMA) method. Dendrograms were plotted using NTSYS-pc 2.10 software (Rohlf 1998). Interpopulation structure among the 5 sites was investigated using Bayesian clustering with STRUCTURE software version 2.3.4 (Pritchard et al. 2000) by testing 10 independent runs for a given number of inferred clusters K, from K = 1 to 8. STRUCTURE runs consisted of 500 000 MCMC generations, after a burn-in period of 100 000 iterations with LOCPRIOR model described by Hubisz et al. (2009). We used the admixture ancestry model under the correlated allele frequency model (Falush et al. 2003). For the optimal value of K in the studied populations, we used the STRUCTURE HARVESTER website (Earl and Vonholdt 2012) to apply the Evanno method (Evanno et al. 2005). The 10 runs of the best K were averaged and visualized with the web application Pophelper (Francis 2017).

### cpDNA trnL-trnF sequence analysis

We chose trnL-trnF cp DNA primer (Table 3) formerly proved to be variable in molecular phylogenetic study of *Plantago* L. (Plantaginaceae) (Taberlet et al. 1991; Rønsted et al. 2002). PCR amplifications were performed using 24.5-μL reactions mixture containing 1 μL genomic DNA (20–30 ng), 18.7 μL Milli-Q ultrapure water (Merckmillipore, Billerica, MA, USA), 2.5 μL (10 ×) Dream Taq Green PCR buffer (ThermoFisher, Waltham, MA, USA), 0.5 μL (10 mM) dNTP mix (ThermoFisher), 1 μL (2.5 mM) MgCl2, 0.5 μL (10 mM) of each primer (Biocenter Kft., Szeged, Hungary), 0.25 μL (1%) BSA (ThermoFisher), 0.5 μL (2%) DMSO (Reanal, Budapest, Hungary), and 0.05 μL (0.5 unit) Dream Taq Green DNA polymerase (ThermoFisher). The thermocycling conditions were as follows: initial denaturation step at 94 ◦C for 5 min, followed by 35 cycles of 30 s of denaturation at 94 °C, 53 °C for 40 s and 2.5 min of extension at 72 °C, with a final extension step at 72 °C for 7 min. The PCR products were applied on a 1.5% (w/v) ethidium bromide stained agarose gel in 1xTBE buffer. PCR products were separated for 40 min at 100 V. Amplified products were purified using a CleanSweep PCR Purification (Applied Biosystems, Waltham, MA, USA) kit according to the manufacturer’s insctructions. Cleaned products were sequenced from both directions using an ABI PRISM 3100 Genetic Analyzer automated DNA sequencer. The trnL-trnF sequences were aligned with *Plantago* sequences from the GenBank (Table 4). *Plantago alpina*, *P. lanceolata*, *P. major* and *P. media* voucher specimens were used as outgroups in the haplotype analyses. *P. alpina* and *P. lanceolata* were chosen from a different subgenus for a greater attributable difference, while *P.media* and *P. major* were from the same subgenus as *P. maxima*, in order to test the differences among species of the same subgenus. The expected genetic similarity among species was high, highest compared to the species from the same subgenus as *Plantago maxima*.

**Table 3:** Description and amplification conditions of cpDNA primer pair used for the molecular study

Name	Forward/Reverse	Sequence (5’ → 3’)	*T*a (°C)
trnL5^’UAAF^	F	CGAAATCGGTAGACGCTACG	53
trnF^GAA^	R	ATTTGAACTGGTGACACGAG	

*Ta* annealing temperature

**Table 4:** Sequence information for *Plantago* species retrieved from GenBank

Species	GenBank accession number	Similarity (%)	Country and origin	Voucher information
*Plantago alpina* L.	AY101932.1	94.03	Origin unknown, cult. (University of Copenhagen)	Jensen et al. 1996
*Plantago lanceolata* L.	AY101952.1	91.39	Origin unknown, cult. (University of Copenhagen)	Rønsted 33 (University of Copenhagen)
*Plantago major*	AY101917.1	98.09	Sachsen-Anhalt (Martin-Luther-University, Halle)	Rønsted 41 (University of Copenhagen)
*Plantago maxima*	MK487969.1	99.40	No data available	Rønsted 28 (University of Copenhagen)
*Plantago media*	AY101920.1	97.40	Origin unknown, cult. (University of Copenhagen)	Rønsted 50 (University of Copenhagen)

### Data analysis for cpDNA trnL-trnF sequence polymorphism

We used BioEdit 7.2.5 software (Hall 1999), to edit the sequence chromatograms, with visual inspection being performed for all polymorphic sites detected. Alignments were performed using ClustalW (Thompson et al. 1994). Information on gaps (indels) within the aligned sequence matrices were coded as binary characters. Gaps were coded following the simple indel coding algorithm (Simmons et al. 2001) using the program FastGap 1.2 (Borchsenius 2009).

PopART (Leigh and Bryant 2015) with implemented Templeton–Crandall–Singh (TCS) statistical parsimony network analysis (Clement et al. 2002) was used to evaluate genealogical relationships among sequences. Each insertion and deletion was considered to be a single mutation event, and all indels were coded as single positions in the final alignments. The connection limit for the TCS analysis was 95% and gaps were treated as a fifth state.

## Results

### Morphometric analyses

**Fig. 2:**
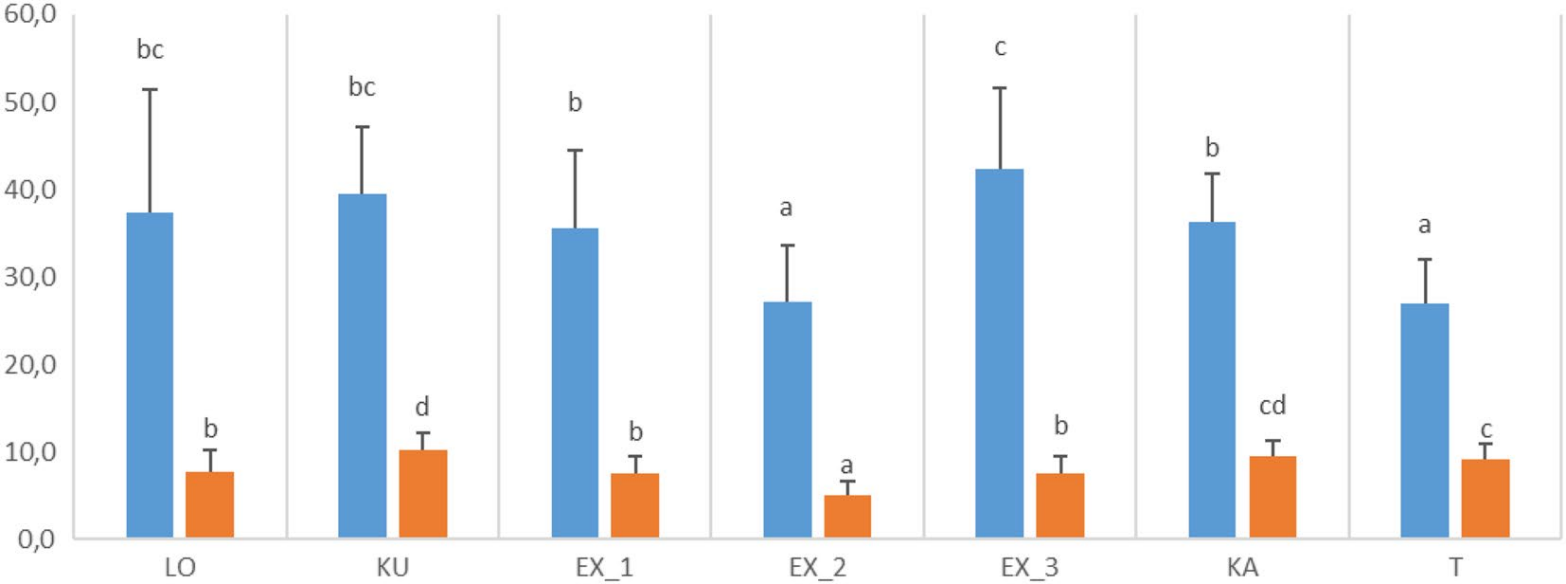
Statistically significant morphometric variables (biggest leaf length and width) of *Plantago maxima* among the Hungarian populations under study, detected by Games-Howell post hoc test (p < 0.05). LO = Táborfalva military shooting range; KU = Kunpeszér; EX = Soroksár Botanical Garden – subpopulation 1,2 and 3; KA = Kakucs; T = Tatárszentgyörgy. The unit of the measurement is cm. Different letters refer to significant differences based on the Games-Howell post hoc tests among the populations

**Fig. 3:**
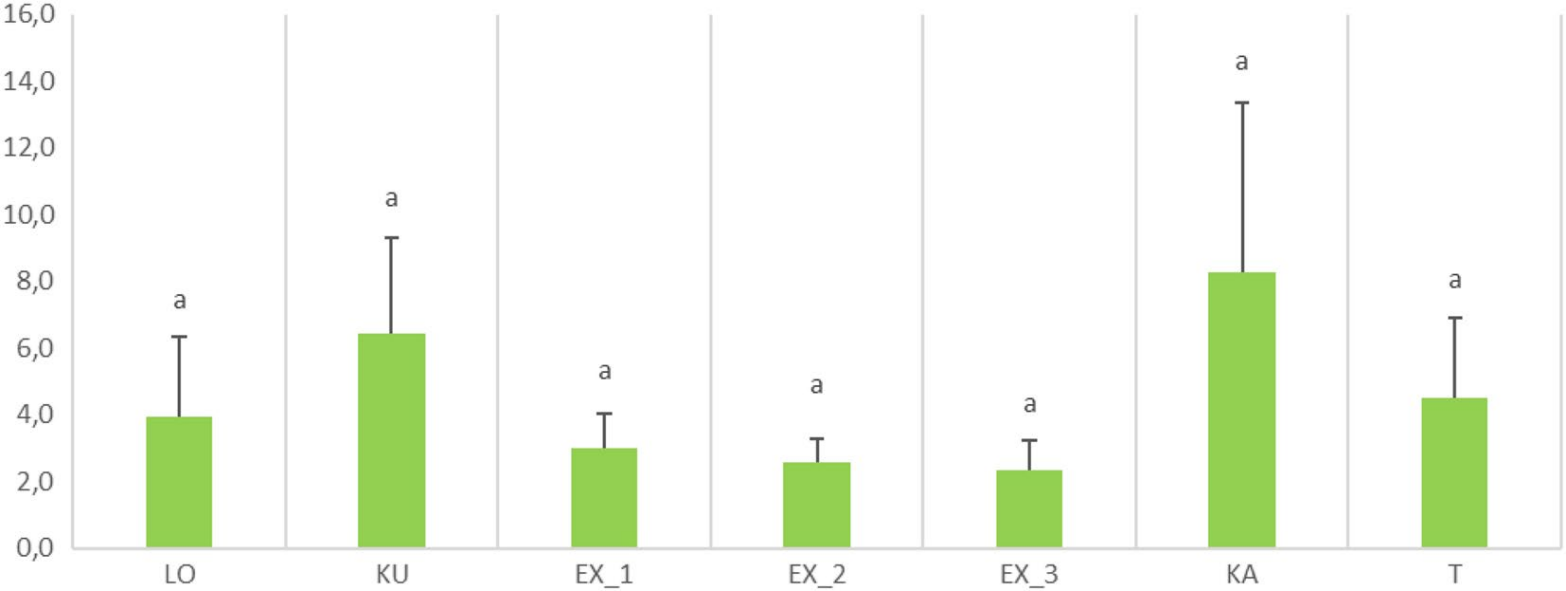
Statistically significant morphometric variables (leaf number) of *Plantago maxima* among the Hungarian populations under study, detected by Tukey post hoc test (p < 0.05). LO = Táborfalva military shooting range; KU = Kunpeszér; EX = Soroksár Botanical Garden – subpopulation 1,2 and 3; KA = Kakucs; T = Tatárszentgyörgy. Different letters refer to significant differences based on the Tukey post hoc tests among the populations

MANOVA yielded significant differences between populations (Wilk’s lambda = 0.32, p < 0.001), with significant intercept (Wilk’s lambda = 0.03, p < 0.001), and significant difference between the studied years (Wilk’s lambda = 0.7, p < 0.001). In case of the biggest leaf length (F(6;375) = 36.32;p < 0.001) and biggest leaf width (F(6;375) = 45,10;p < 0.001) there were significant differences between the populations (Fig. 2). However in case of the leaf number, no significant differences were detected (F(6;375) = 1.961; p = 0.07) (Fig. 3). According to the observed traits ex situ subpopulations fit into the values experienced in natural populations.

**Fig. 4:**
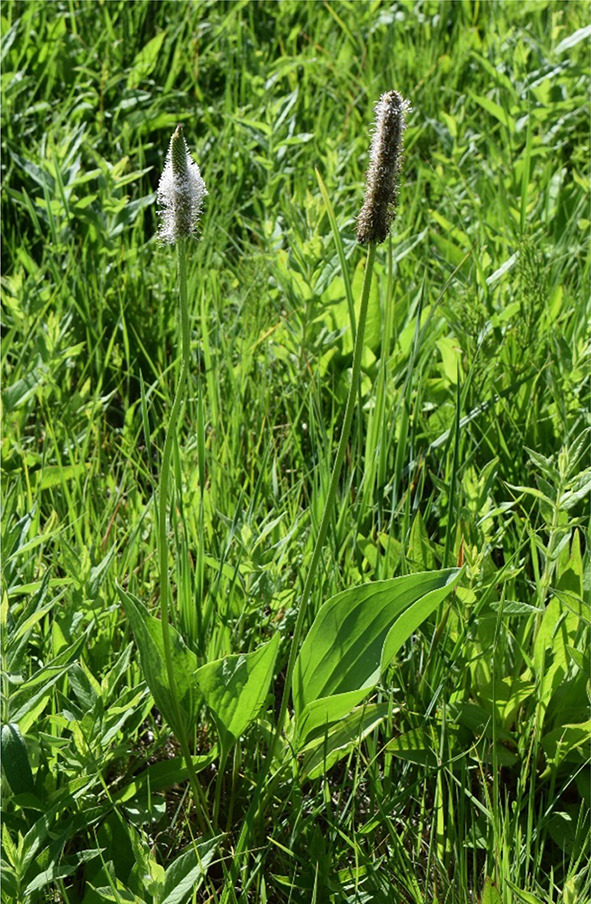
*Plantago maxima* individuals flowering in the ex situ collection

Individuals reached generative phase (Fig. 4) only at subpopulation 1. In 2019 two individuals, in 2020 three individuals, in 2021 four individuals produced flowers.

### Chromosome count and FISH

**Fig. 5:**
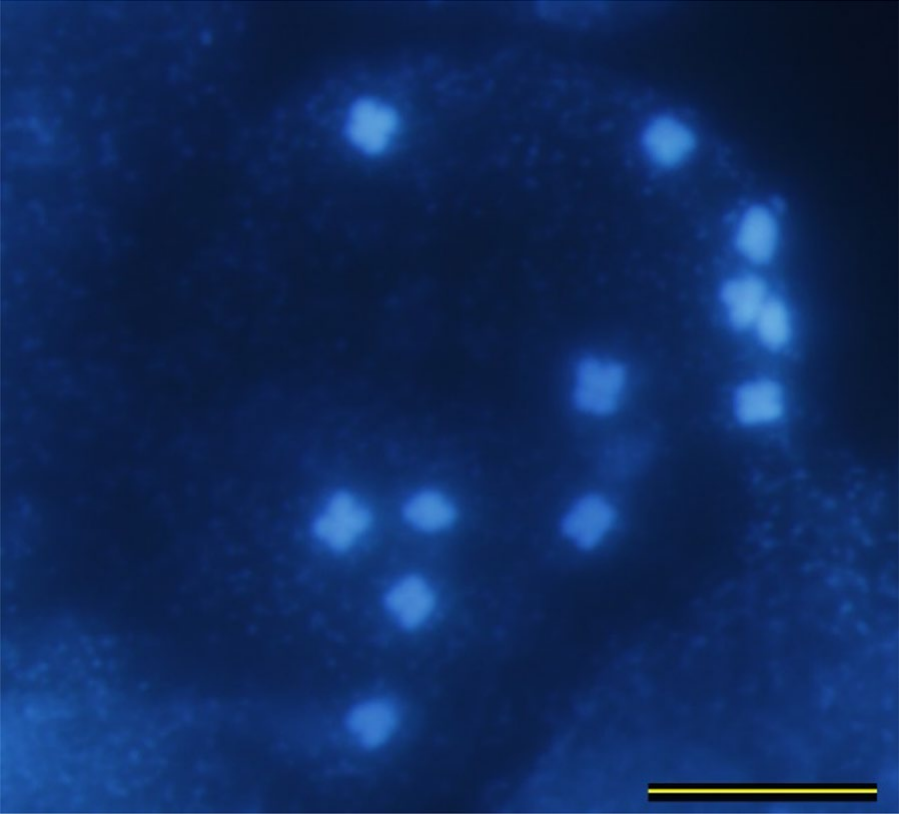
Mitotic metaphase of *Plantago maxima* after staining with (DAPI) (2*n* = 2*x* = 12.). Scale bar = 10 μm

**Fig. 6:**
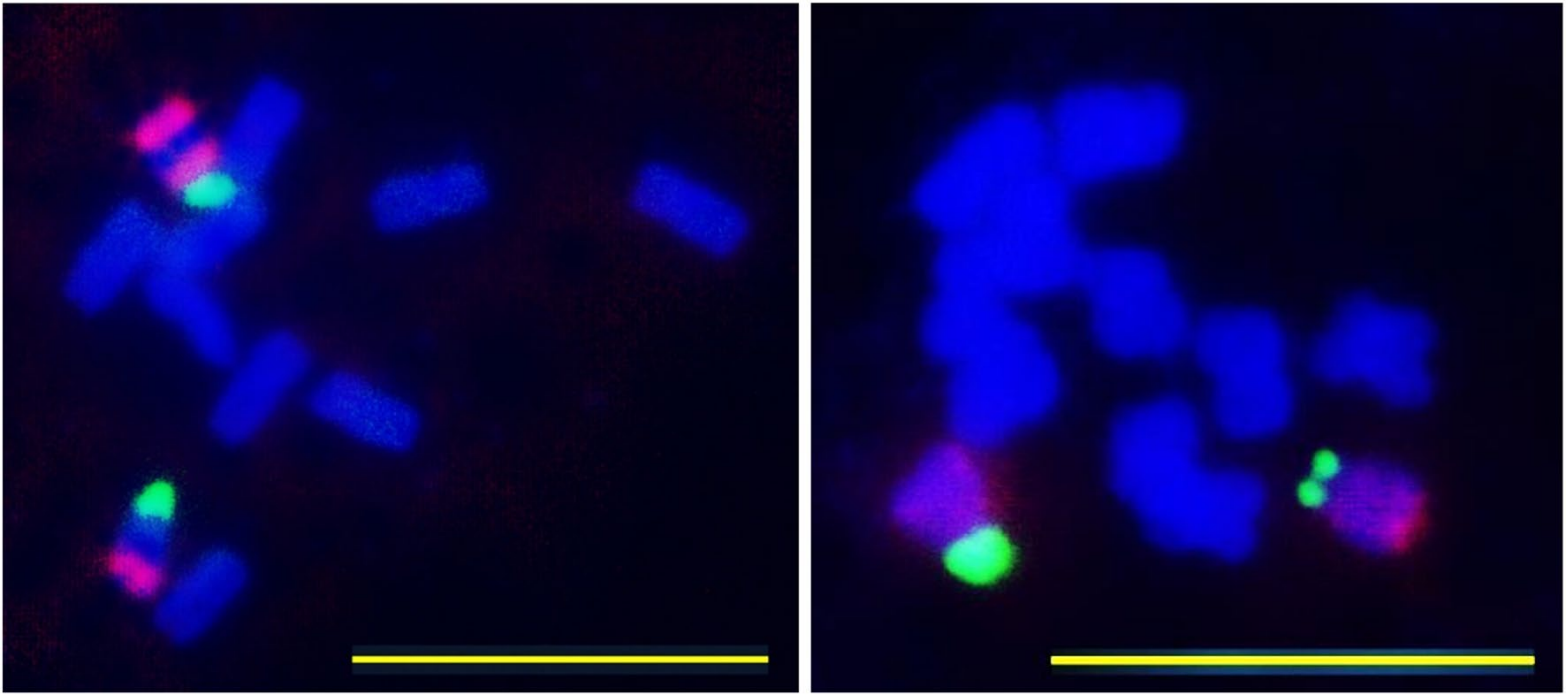
FISH mapping of 35S (green) and 5S rDNA (red) on somatic metaphase chromosomes of *Plantago maxima* (2*n* = 2*x* = 12). Scale bar = 10 μm

**Fig. 7:**
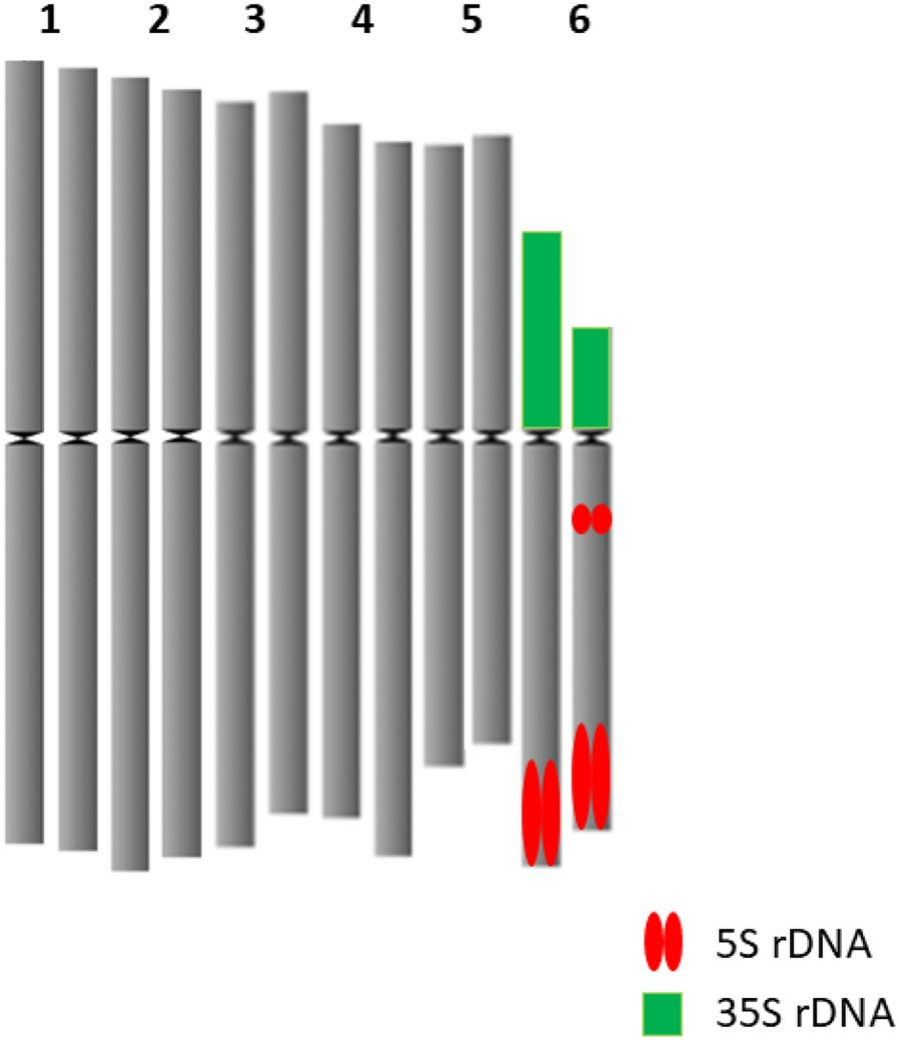
Idiogram of *Plantago maxima*

The karyotype formula of *P. maxima* is 5 m + 1a. Chromosomal localization of 35S and 5S rDNA loci have been investigated in two individuals belonging to two Hungarian populations (Kakucs) and (Kunpeszér). At least five metaphases were investigated per individual. One 35S and two 5S rDNA loci were positioned on the acrocentric chromosome 6. 35S rDNA was located subterminally on the short arm, while one 5S rDNA loci were located proximally to the centromere and the other subterminally on the long arm. 35S rDNA locus was heteromorphic. 5S rDNA locus positioned proximally to the centromere was at the edge of visibility. The mitotic metaphase, FISH and idiogram is presented in Figs. 5, 6 and 7.

### Genetic diversity and differentiation

The amplification of the ISSR fragments in the 100 individuals, analysed with eight primers, found variable after a primary test, yielded 100 unambiguous and reproducible electrophoretic bands (Additional file 1: Figs. S1 and S2) ranging from seven to 16 bands for each of the primers. Eighty-seven bands (87.0%) were polymorphic when comparing all the samples (Table 5).

**Table 5:** Polymorphism indices for inter simple sequence repeats (ISSR) primers used for genetic diversity analysis in the *Plantago maxima* study

Primer name	Band size range (bp)	Total bands	Polymorphic bands	Polymorphism (%)
UBC 807	520–1270	13	12	92.31
UBC 808	480–1240	15	12	80.00
UBC 811	640–1450	13	11	84.62
UBC 857	280–790	9	4	44.44
UBC 835	420–760	7	6	85.71
UBC 816	680–2000	16	15	93.75
UBC 818	420–990	11	11	100
UBC 809	700–1585	16	16	100
Mean	280–2000	100	87	87

The percentage of polymorphic loci was 69–85% between the populations. The highest polymorphism was detected in Kunpeszér population, where two private bands were found. Nei’s gene diversity was lower in Kakucs and in the ex situ collection (H_e_ = 0.206), while this value was the highest in Kunpeszér (H_e_ = 0.257). The values of Shannon’s information index reflect similar results, the lowest in ex situ collection, the highest 0.396 in Kunpeszér population (Table 6). AMOVA analysis revealed significant (p < 0.001) differences within the populations. Of the total genetic diversity, 8% was attributable to among populations and the remaining 92% to within populations (Table 7).

**Table 6:** Genetic variation in populations of *Plantago maxima* detected by inter-simple sequence repeat (ISSR) markers

ID	*N*	n	PPL	N_a_	N_e_	I	H_e_(S.E.)	uH_e_
EX	20	69	69.00	1.610	1.337	0.317	0.206 (0.018)	0.211 (0.019)
T	20	74	74.00	1.680	1.365	0.344	0.223 (0.018)	0.229 (0.019)
KA	20	72	72.00	1.670	1.337	0.320	0.206 (0.018)	0.212 (0.019)
KU	20	85	85.00	1.850	1.422	0.396	0.257 (0.017)	0.264 (0.018)
LO	20	73	73.00	1.650	1.338	0.324	0.209 (0.018)	0.214 (0.018)
Mean values		74.60	74.60	1.692	1.360	0.340	0.220 (0.008)	0.226 (0.008)

*N* sample size, *n* number of polymorphic loci, *PPL* percentage of polymorphic loci, *N*a observed mean number of alleles per locus, *N*e effective mean number of alleles per locus, *I* Shannon’s information index, *H*e Nei’s gene diversity, *S.E.* standard error, *uH*e Unbiased expected heterozygosity, ID abbrev. EX - Soroksár Botanical Garden, T - Tatárszentgyörgy, KA - Kakucs, KU - Kunpeszér, LO - Táborfalva military shooting range

**Table 7:** Summary of the analysis of molecular variance (AMOVA) in giant plantain using GenAlEx

Source of variation	d.f	Sum of Squares	Estimated Variance	% Total Variance	Φ	Significance (*p*)
Among populations	4	140,920	1130	8	0.082	< 0.001
Within populations	95	1,199,000	12,621	92		
Total	99	1,339,920	13,752	100		

*Φ* PhiPT value

### Population genetic structure

**Fig. 8:**
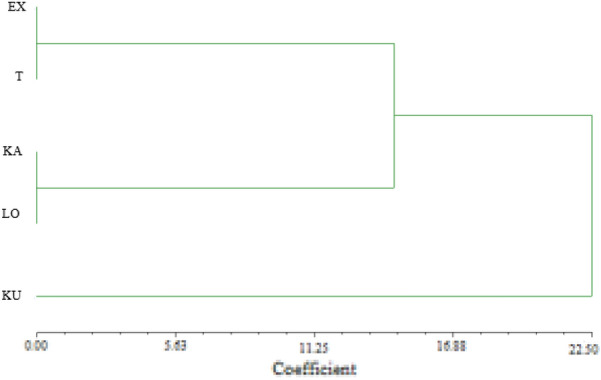
Dendrogram of the 5 giant plantain populations studied generated by UPGMA cluster analysis of the similarity matrix obtained using Nei’s genetic distance based on ISSR data (Nei, 1978)

The UPGMA-based dendrogram based on a similarity matrix using Jaccard’s coefficients is shown in Additional file 1. Genetic relationships among the studied habitats were calculated (Table 8) and an UPGMA-based dendrogram obtained is shown in Fig. 8. and Additional file 1: Fig. S3. The five giant plaintain populations were grouped into three subgroups. One clade included ex situ and Tatárszentgyörgy, while the second clade included Kakucs and Lőtér. The third clade represented only the Kunpeszér population, which was divergent from the other populations.

**Table 8:** Pairwise population matrix of Nei’s genetic distance of the giant plantain populations based on 8 ISSR markers

EX	T	KA	KU	LO	
0.000					EX
0.038	0.000				T
0.025	0.036	0.000			KA
0.050	0.048	0.041	0.000		KU
0.033	0.040	0.025	0.041	0.000	LO

**Fig. 9:**
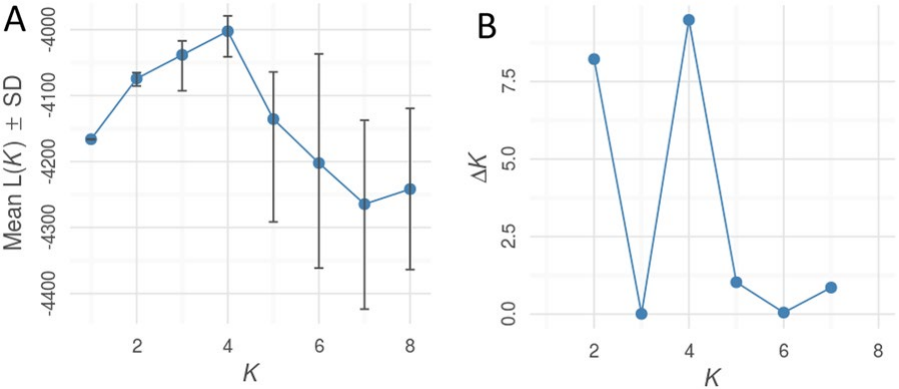
STRUCTURE HARVESTER results for *Plantago maxima* populations. **A** Mean of lnK probability and **B** delta K

**Fig. 10:**
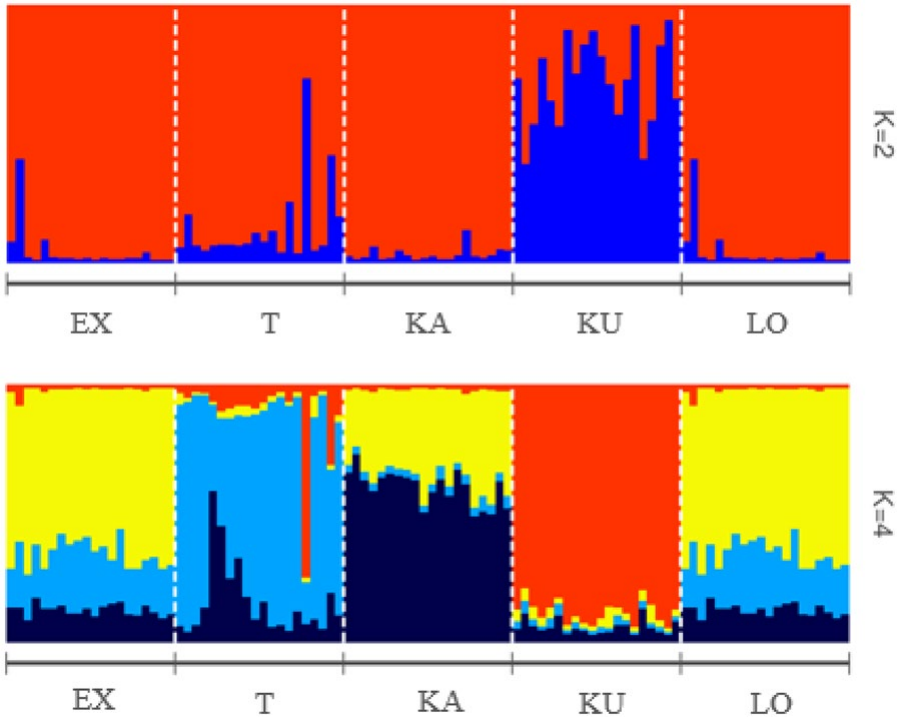
Estimated genetic population structure of *Plantago maxima* populations based on ISSR markers for K = 2 and K = 4 revealed STRUCTURE with the location priory model. EX, Soroksár Botanical Garden – ex situ; TAT, Tatárszentgyörgy; KAK,Kakucs; KUN, Kunpeszér; LO, Táborfalva military shooting range

STRUCTURE analysis on the ISSR dataset revealed the highest DK for K = 4 (DK = 8; Fig. 9). Admixed individuals among clusters were observed (Fig. 10). STRUCTURE analyses clearly discriminated the Kunpeszér population and classified into an individual cluster. This result is even more clear when K = 2 is considered. The remaining populations show a greater level of admixture and mixed population structure.

### cpDNA sequence trnL-trnF

**Fig. 11:**
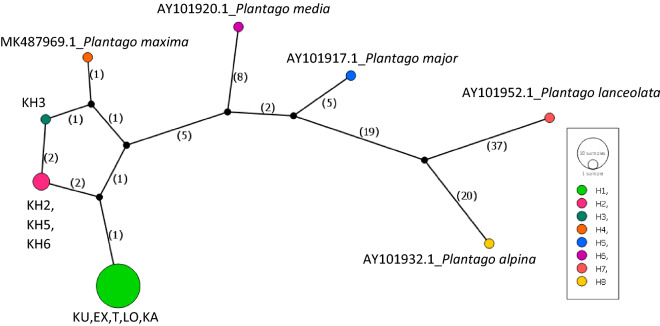
The haplotype network obtained based on Templeton–Crandall–Singh analysis of the trnL-trnF cpDNA region of *Plantago* samples. Black dots indicate missing intermediate haplotypes that were not observed in the analyzed sample set. The numbers on branches represent mutation steps (number of base pair changes) between haplotypes. For abbreviation see Table 1.

The total length of the *trnL-trnF* sequence was 837 bp. The haplotype network based on the *trnL-trnF* region consists of four *Plantago maxima* haplotypes (Fig. 11). H5-H8 haplotypes represent different *Plantago* species, while H1-H4 represent *Plantago maxima* haplotypes. The Hungarian populations were not polymorphic, sharing the same haplotype across individuals and populations in the study region (H1). Between the Hungarian and Kazakhstan samples the difference occurred in two positions: mononucleotide (A) microsatellite in 226–236 bp position, and also a mononucleotide (T) microsatellite in 589–597 bp region. The Kazakhstan population comprised two different haplotypes (H2-H3) based on the two microsatellite regions length. The *Plantago maxima* sample from NCBI (of unknown origin) represented another haplotype with two SNP in the 38 and 247 bp positions (H4).

## Discussion

The Pannonian Basin holds a great botanical value providing primary and secondary refugia for European steppe species (Willner et al. 2021). *Plantago maxima* is one of the remaining relict steppe plants in the Hungarian flora. The present study is the first to examine the genetic structure and population variability of in situ and established ex situ giant plantain populations. To plan future conservation actions, it is important to analyse the current state of the populations and to compare the naturally occurring diversity to the established ex situ collection.

The results of a comparative morphological trait-based analysis showed that the garden population fits into the trait variation of the natural populations. During a long-term conservation period, adaptation to the novel environment can induce morphological changes and shifts in life-history traits (Hammer 1984; Ensslin and Godefroid 2019). Changes can occur even in a short period of time as Rauschkolb et al. (2019) reported in their study. Therefore, it is important to evaluate the changes after the establishment of the ex situ populations. Volis and Blecher (2010) suggests establishing of new collections in near-natural habitat where adaptation process to novel environment can be eliminated. Our trait-based results suggest that *Molinia* meadow of the Soroksár Botanical Garden was a good choice for preserving ex situ collection. Habitat conditions are very similar to that experienced in the natural sites and this most probably helps reducing the chance of local adaptation to garden conditions.

During the three years observation period most of the individuals of the ex situ collection remained in vegetative state, the first few flowering individuals appeared in 2019, in just one of the subpopulations. However, the occurrence of the generative phase indicates the stabilization of the population in the new environment, which is essential for long-term preservation. In case of *Plantago coronopus* the initiation of flowering was stage-dependent and after the development of 14 leaves, plants started to form flowering buds (Koelewijn 2004). In case of *Plantago maxima*, as no significant differences were observed between the natural populations and ex situ subpopulations it is more likely that the delay of flowering-phase is caused by the differences in nutrient levels or different competition regimes within the new ex situ habitat. However, this statement needs further investigation.

Genetic studies are important to evaluate natural population diversity and guide conservation efforts. To the best of our knowledge this is the first report on chromosome features and rDNA localization of *Plantago maxima*. All individuals from the four Hungarian populations were diploid (2*n* = 2x = 12) as Soó (1970) reported previously. The characterization and physical mapping of rDNA sites in *Plantago* resulted in species-specific patterns (Dhar et al. 2006, 2017; Wong and Murray 2014). Each *Plantago* species investigated so far has unique rDNA pattern enabling discrimination of species according to the rDNA loci position. *Plantago* exhibit accelerated structural evolution of their plastomes and highly accelerated substitution rates throughout the genome, which also explains the unique rDNA localization among different Plantago species (Cho et al. 2004; Mower et al. 2021).

ISSRs are widely and successfully used in evaluating the genetic diversity of natural populations and so are important tools to guide conservation efforts and support ex situ collection establishment. ISSR markers are highly variable, therefore using a greater sample size and an extensive statistical analysis is required for better resolution. When comparing the in situ and ex situ populations it is important to evaluate the genetic representativeness especially of the established ex situ collection. In the Hungarian populations *Plantago maxima* reaches a relatively high genetic diversity (mean value of H_e_ = 0.220), in comparison to other endangered and endemic *Plantago* species where H_e_ = 0.1965–0.2309 was reported using ISSR markers (Ferreira et al. 2013). Higher genetic diversity can be reasoned by additional gene flow between the populations and recent isolation events. The overall equilibrated genetic diversity in the natural populations makes it important to conserve the genetic material of all populations, so the establishment of additional ex situ stocks are desirable in the future. Genetic diversity of in situ populations and ex situ stock was very similar, and in some stands displayed the same values (He = 0.206). In a few studies including different species genetic representation of the natural populations and botanical garden collections were also alike (Etisham-Ul-Haq et al. 2001; Li et al. 2002, 2018; Ensslin et al. 2011; Chen et al. 2013; Guimaraes et al. 2019). For example a study revealed similar genetic diversity between wild and garden stock plants in *Leucothrinax morrisii* (H.Wendl.) C. Lewis and Zona (Namoff et al. 2010). The mean diversity (H_e_) was similar in the ex situ collection and wild populations. Also a great proportion of genetic variability was preserved in the ex situ population of *Sinocalycanthus chinensis* W. C. Cheng and S. Y. Chang (Chen et al. 2013).

However, a great number of studies highlighted the underrepresentation of genetic variability in garden collections (Rucińska and Puchalski 2011; Lauterbach et al. 2012; Brütting et al. 2012; Christe et al. 2014; Miao et al. 2015; Wilson et al. 2017; Li et al. 2018; Chacón-Vargas et al. 2019). For instance reduced genetic diversity in ex situ collection was observed in case of *Cochlearia polonica* E. Frohlich (Rucińska and Puchalski 2011). Maintaining high genetic diversity is essential to eliminate negative trends in the genetic make-up of the ex situ collections.

The genetic structuring of natural plant populations results from the distribution shifts, habitat fragmentation, selection, gene flow and genetic drift or mutation events (Hamrick and Schnabel 1985; Schaal et al. 1998). The UPGMA cluster analysis revealed that the Kunpeszér population is differentiated from the other four studied population. Similarly STRUCTURE analysis revealed also that Kunpeszér population which is the largest natural population in Hungary differentiated from the others in case of K = 4. A great level of admixture and mixed population structure was observable among the remaining populations. The AMOVA results revealed that most of the diversity occurs within populations (92%), which is in accordance with STRUCTURE results. The low population differentiation can be due to small geographic distances among the observed populations as it was observed in the westernmost German exclave of *Adonis vernalis* (Kropf et al. 2020). The Hungarian populations have a small distribution area, also some populations have small number of individuals. The above mentioned reasons could explain the lower diversity value and highlights the importance of ex situ conservation.

The trnL(UAA)-trnF(GAA) region was formerly successfully used in a molecular phylogenetic study of *Plantago* L. (Plantaginaceae) (Rønsted et al. 2002). Based on the cpDNA trnL-trnF marker, a study with *Plantago brutia* and *Plantago media* revealed the occurrence of several haplotypes of both species from Sweden to the Iberian Peninsula and the Balkans (Palermo et al. 2010). However, no sequence variation was detected within the Hungarian populations. A low haplotype diversity at the edge populations were also observed in several studies (Becker 2005; Hensen et al. 2010; Durka et al. 2013; Kropf et al. 2020) indicating possible recent population declines. However, it is important to highlight the presence of a distinct haplotype preserved in the Hungarian populations, compared to the Kazakhstan population originating from the central part of the species’ distribution area. Further studies and more samples should be analysed to estimate the distance between Central-Asian and European populations.

Unique haplotypes were also observed in edge populations of *Poa badensis* (Plenk et al. 2017) and *Adonis vernalis* (Kropf et al. 2020). Peripheral populations are often smaller in population size and genetically more differentiated, frequently with lower genetic diversity compared to central populations (Sagarin and Gaines 2002; Eckert et al. 2008; Sexton et al. 2009). However, there are several exceptions, when edge populations do not support abundant centre hypothesis (Abeli et al. 2014). These results indicate the great importance to conserve Hungarian populations for preserving species natural gene stock on the periphery.

## Conclusions

We studied the morphological, cytological and genetic diversity of the natural populations and ex situ botanic garden collection of the highly protected *Plantago maxima* in Hungary. These populations at the westernmost periphery of species’ distribution range represent an important gene pool that is shown to be different and adaptively diverge from central populations. There were no observable morphological changes in the garden populations seven years after introduction compared to the wild populations. This result highlights that since the establishment of the ex situ stock no maladaptive changes have occurred. This is an important prerequisite for successful conservation and restoration. Long-term conservation however requires further monitoring activity. We found only four flowering ex situ individuals, but it would be desirable to have more. Therefore, the assessment of the needs for flowering requires further studies.

Based on the chromosome study there were no observable differences between the Hungarian populations, all studied individuals were diploids exhibiting same chromosome number. Accordingly in the establishment of the ex situ collections no special attention should be drawn to population ploidy level. It would be desirable to compare our findings with other populations from the central area to evaluate possible differences in the central-peripherial context.

By evaluating genetic variability of the natural populations in comparison with ex situ stock we could not detect decrease of genetic variability within later as genetic indexes were fairly similar. This proportion of variation indicates that the ex situ collection bears high adaptive potential for even a longer conservation period. However, the different genetic structuring suggests a possible loss of naturally occurring alleles (Kakucs n = 95) from the ex situ population (n = 92), therefore introduction of new individuals is required to strengthen the botanical garden population. Kunpeszér population bears the most elevated genetic diversity, therefore the establishment of new ex situ collection with propagules originating from that population is highly recommended. The lower number of individuals in Táborfalva and Tatárszentgyörgy preserving similar genetic diversity in these two localities also requires the need of establishing new ex situ gene stocks.

## Supplementary Information

**Additional file 1: Fig. S1.** ISSR banding profile of *Plantago maxima *individuals with UBC 808 primer. **Fig. S2.** ISSR banding profile of *Plantago maxima *individuals with UBC 818 primer. **Fig. S3.** Cluster analysis among the individuals of *Plantago maxima *based on ISSR molecular marker.

## Data Availability

The datasets used and/or analysed during the current study are available from the corresponding author on reasonable request.
