# Supplementary material for: Living on the edge: morphological, karyological and genetic diversity studies of the Hungarian Plantago maxima populations and established ex situ collection
Source: Bot Stud. 2023 Jan 24;64:2. doi: 10.1186/s40529-022-00365-6 (PMC9873897; doi:10.1186/s40529-022-00365-6)
Supplement: Supplementary file 1 — Additional file 1: Fig. S1. ISSR banding profile of Plantago maxima individuals with UBC 808 primer. Fig. S2. ISSR banding profile of Plantago maxima individuals with UBC 818 primer. Fig. S3. Cluster analysis among the individuals of Plantago maxima based on ISSR molecular marker. [file 40529_2022_365_MOESM1_ESM.docx]

Additional file 1

Figure/Material


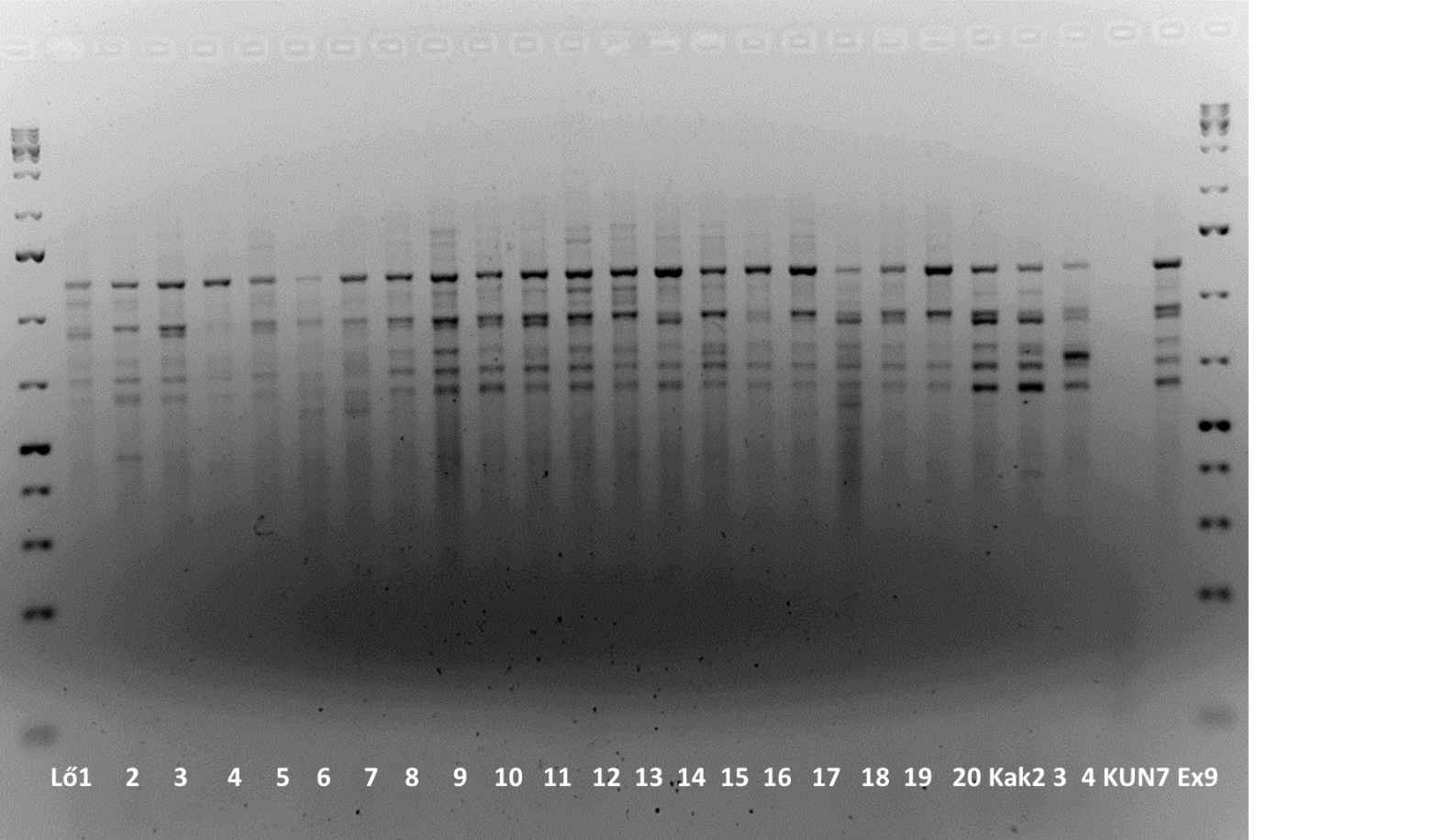


Fig. S1. ISSR banding profile of *Plantago maxima* individuals with UBC 808 primer.


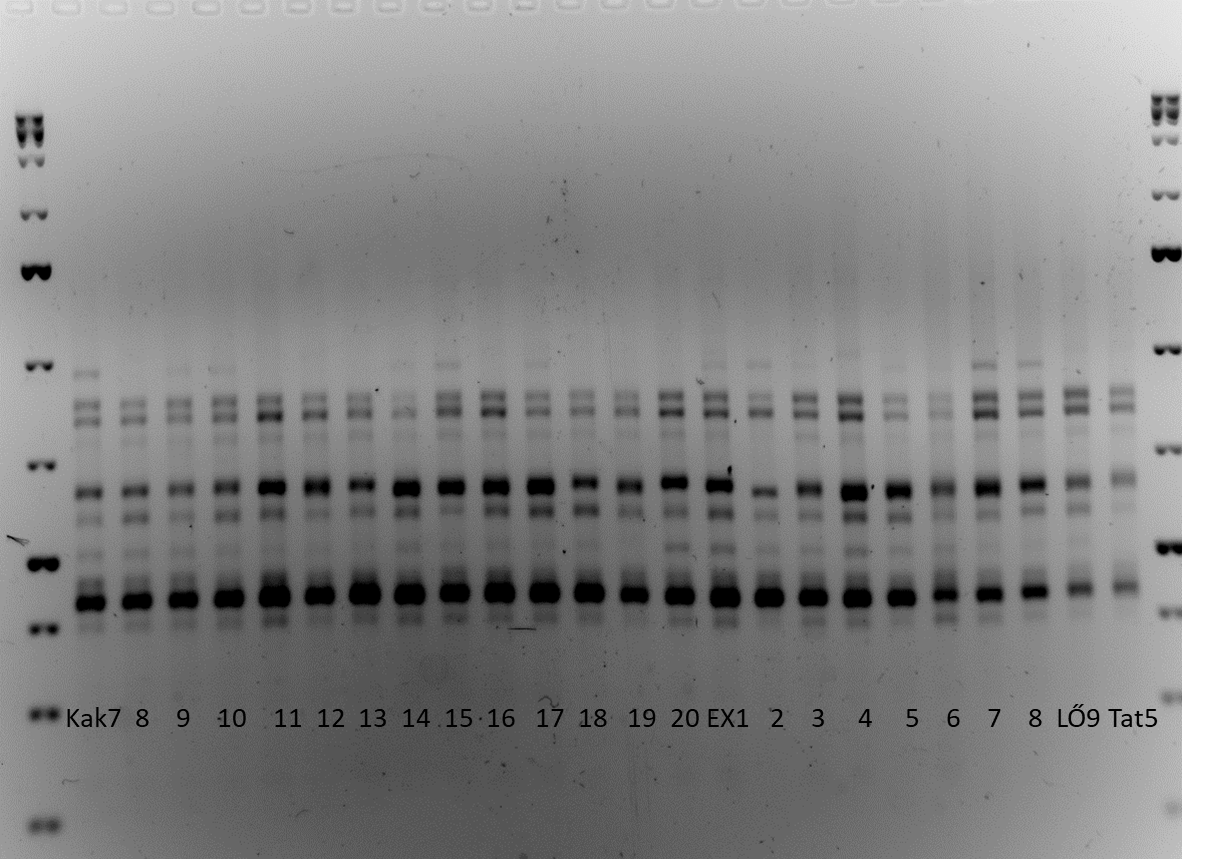


Fig. S2. ISSR banding profile of *Plantago maxima* individuals with UBC 818 primer


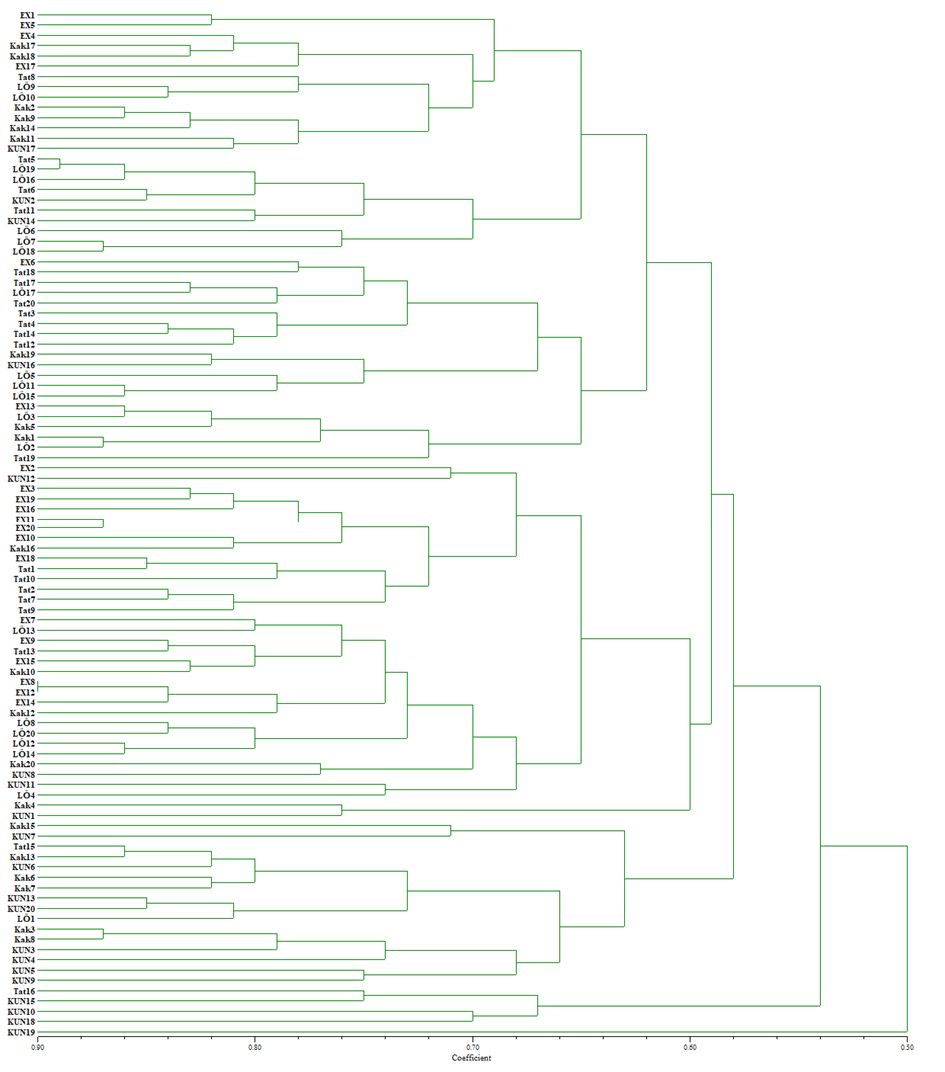


Figure S3. Cluster analysis among the individuals of *Plantago maxima* based on ISSR molecular marker
